# 3.4 NEURAL OSCILLATIONS AND EXCITATION/INHIBITION BALANCE IN SCHIZOPHRENIA: A DEVELOPMENTAL PERSPECTIVE

**DOI:** 10.1093/schbul/sby014.006

**Published:** 2018-04-01

**Authors:** Peter Uhlhaas

**Affiliations:** Institute of Neuroscience & Psychology, University of Glasgow

## Abstract

**Background:**

Schizophrenia (ScZ) is a neurodevelopmental disorder that characteristically emerges during the transition from adolescence to adulthood. However, the mechanisms that underlie the expression of psychotic symptoms and cognitive deficits during this developmental period are still unclear. In my presentation, I will summarize data from EEG/MEG-work that has investigated the maturational changes in in neuronal dynamics during adolescence as well as the possibility that aberrant rhythmic activity is present in clinical high-risk participants.

**Methods:**

A sample of participants meeting CHR-criteria (n=100) from the ongoing Youth Mental Health Risk and Resilience (YouR) Study and 50 matched controls were recruited as well as a sample of n = 20 participants meeting first-episode psychosis (FEP) criteria. We examined auditory and visual-induced oscillations as well as resting-state Magnetoencephalographical (MEG)-data and obtained estimates of spectral power and phase-synchronization at source-level. MEG-recordings were accompanied by Magnetic Resonance Spectroscopy (MRS) measurements of GABA and Glutamate-levels in auditory and visual cortices.

In addition, we examined the development of neural oscillations in a sample of n = 100 children and adolescents (age range: 12–21 years) during a working memory task and during spontaneous activity to identify critical periods for the development of neural dynamics.

**Results:**

CHR-participants were significantly impaired in the generation of both auditory and visual gamma-band oscillations as well as characterized by an increase in broad-band, resting-state gamma-band power. The latter points towards an increase in excitability-levels of neural circuits which is supported by increased Glutamate-levels in sensory regions while GABA-levels were not different from controls. Similar patterns in both MEG- and MRS-parameters were observed in the FEP-group. Finally, our developmental data highlight that the transition from adolescence to adulthood is characterized by profound changes in both amplitude and synchrony dynamics, highlighting the possibility that critical period mechanisms that underlie the expression of psychosis are impaired in ScZ.

**Discussion:**

Together, these data indicate that aberrant neural oscillations in ScZ highlight the crucial contribution of impaired neural dynamics that are likely to result from dysfunctional Excitation/Inhibition balance parameters. Moreover, the onset of schizophrenia during the transition from adolescence to adulthood suggests that critical period mechanisms that support the expression of high-frequency oscillations are impaired.

